# Head to Head Comparison of Short-Term Treatment with the NAD^+^ Precursor Nicotinamide Mononucleotide (NMN) and 6 Weeks of Exercise in Obese Female Mice

**DOI:** 10.3389/fphar.2016.00258

**Published:** 2016-08-19

**Authors:** Golam M. Uddin, Neil A. Youngson, David A. Sinclair, Margaret J. Morris

**Affiliations:** ^1^Department of Pharmacology, School of Medical Sciences, University of New South Wales, Sydney, NSWAustralia; ^2^Department of Genetics, Paul F. Glenn Center for the Biology of Aging, Harvard Medical School, Boston, MAUSA

**Keywords:** nicotinamide mononucleotide, high fat diet, treadmill exercise, C57BL6 female mice, mitochondria, liver, muscle

## Abstract

Obesity is well known to be a major cause of several chronic metabolic diseases, which can be partially counteracted by exercise. This is due, in part, to an upregulation of mitochondrial activity through increased nicotinamide adenine dinucleotide (NAD^+^). Recent studies have shown that NAD^+^ levels can be increased by using the NAD^+^ precursor, nicotinamide mononucleotide (NMN) leading to the suggestion that NMN could be a useful intervention in diet related metabolic disorders. In this study we compared the metabolic, and especially mitochondrial-associated, effects of exercise and NMN in ameliorating the consequences of high-fat diet (HFD) induced obesity in mice. Sixty female 5 week old C57BL6/J mice were allocated across five groups: Chow sedentary: CS; Chow exercise: CEX; HFD sedentary: HS; HFD NMN: HNMN; HFD exercise: HEX (12/group). After 6 weeks of diet, exercise groups underwent treadmill exercise (15 m/min for 45 min), 6 days per week for 6 weeks. NMN or vehicle (500 mg/kg body weight) was injected (i.p.) daily for the last 17 days. No significant alteration in body weight was observed in response to exercise or NMN. The HFD significantly altered adiposity, glucose tolerance, plasma insulin, NADH levels and citrate synthase activity in muscle and liver. HEX and HNMN groups both showed significantly improved glucose tolerance compared to the HS group. NAD^+^ levels were increased significantly both in muscle and liver by NMN whereas exercise increased NAD^+^ only in muscle. Both NMN and exercise ameliorated the HFD-induced reduction in liver citrate synthase activity. However, exercise, but not NMN, ameliorated citrate synthase activity in muscle. Overall these data suggest that while exercise and NMN-supplementation can induce similar reversal of the glucose intolerance induced by obesity, they are associated with tissue-specific effects and differential alterations to mitochondrial function in muscle and liver.

## Introduction

Obesity is a global health issue with increasing prevalence. According to the WHO (2015), 1.9 billion adults were overweight globally in 2014, of which 0.6 billion were obese. In 2014, The McKinsey Global Institute reported that if the current growth rate continues, by 2030, almost half of the world’s adult population will be overweight or obese. The increasing prevalence of obesity leads to an increased risk of several other non-communicable diseases including type 2 diabetes, cardiovascular disease, some cancers, respiratory conditions, fatty liver disease, reproductive disorders, depression, and other mental health conditions (Gonçalves et al., 2012; Morris et al., 2014). Furthermore recent studies on maternal obesity have revealed the alarming metabolic impact on offspring, including increased risk for obesity (Borengasser et al., 2014; Catalano, 2015; Raipuria et al., 2015), highlighting the importance of testing interventions in obese females.

Primarily, the obesity epidemic can be explained as a result of energy imbalance. Increased energy rich food intake and/or decreased physical activity result in increased adiposity (Berthoud et al., 2011; WHO, 2015). Therefore diet is the most commonly used intervention for obesity. A variety of human and animal studies have suggested that exercise can exert health benefits in obesity (Christ et al., 2002; Flores et al., 2006; Vieira et al., 2009; Ross et al., 2015).

Mitochondria play a vital role in cellular functions such as regulation of energy metabolism, ATP generation, and calcium handling. Cellular energy in the form of ATP, is produced by the interconversion of NAD^+^ and NADH as a part of beta oxidation, glycolysis, the TCA cycle and oxidative phosphorylation. Previous studies have implicated mitochondrial dysfunction such as decreased mitochondrial numbers, lower levels of mitochondrial enzymes, and lower ATP synthesis in muscle and liver as key mechanism mediating obesity-related diseases such as insulin resistance and type 2 diabetes (Pérez-Carreras et al., 2003; Lowell and Shulman, 2005; Kim et al., 2008; Mantena et al., 2008; Jheng et al., 2012; Agil et al., 2015). Consequently interventions that alter mitochondrial biogenesis or function have been proposed as an option for combatting obesity-related disease (Joseph et al., 2011; Holmström et al., 2012).

Though originally considered a house keeping metabolite required for redox reactions, NAD^+^ is now recognized as a central signaling molecule and enzyme cofactor that is involved in a variety of fundamental biological processes including energy metabolism, lifespan regulation, DNA repair, apoptosis, and telomere maintenance (Belenky et al., 2007). NAD^+^ levels in metabolic tissues decrease with age, and also in obesity (Yoshino et al., 2011; Gomes et al., 2013). However, mouse models have shown that physical exercise can increase NAD^+^ levels in metabolic organs and improve mitochondrial biogenesis and function (Cantó et al., 2010; Geng et al., 2010; Koltai et al., 2010). Therefore the beneficial effects of exercise are thought to be partly mediated through increasing NAD^+^. Mechanistically, the Sirtuin class of enzymes are one link between NAD^+^ and improved metabolism as they use NAD^+^ as a cofactor in their regulation of mitochondrial biogenesis and respiration efficiency, and metabolism of fats and carbohydrates (Rodgers et al., 2005; Gerhart-Hines et al., 2007; White and Schenk, 2012).

Nicotinamide mononucleotide (NMN) is a precursor of NAD^+^ biosynthesis. Supplementing mice with NMN can increase NAD^+^ levels and ameliorate glucose intolerance in high fat diet (HFD)-induced diabetes, and aged mouse models (Yoshino et al., 2011; Gomes et al., 2013). Considering that exercise and NMN supplementation both increase NAD^+^ levels, thereby improving metabolism in obese mice, we hypothesized that NMN would induce similar effects on mitochondrial biogenesis and function as exercise. We chose to study females, given the growing obesity rates in women of childbearing age and the effects of maternal obesity on the next generation (Black et al., 2013). Specifically, we examined effects of treadmill exercise and NMN on weight gain, adiposity and glucose tolerance, and measured mitochondrial copy number, and indices of metabolic and mitochondrial function in two key metabolic organs, muscle and liver.

## Materials and Methods

### Animal Experimentation

All animal procedures were approved by the Animal Ethics Committee, UNSW, ethics number 13/25B. Five week old female C57BL6/J mice (*n* = 60) were purchased from Animal Resource Centre, Canning Vale, WA, Australia. Animals were housed (4 mice/cage) at 21°C ± 2°C (12:12 h light/dark) at the Biological Resources Centre facility, UNSW, Australia. Mice were ear punched 2 days after they arrived. A week later groups of mice with similar average body weight were assigned to either a control group (*n* = 24) that was fed standard rodent chow (11 kJ/g, maximum crude fat 4% total food weight from Gordon’s Stock Feeds, Yanderra, NSW, Australia) or an HFD group (*n* = 36). The HFD pellets were a semi-pure high fat diet formulation for laboratory rats and mice based on Research Diets D12451 bought from Specialty Feeds, Glen Forrest, WA, Australia (contains 23.5% of total weight is fat and 19MJ/kg digestible energy, Speciality Feeds SF 04-001). Five weeks after dietary intervention the difference in average body weight between chow and HFD groups was 23%. The mice were then distributed across five different groups; again mice were selected so that the average body weights of the different intervention and control subgroups were similar. The five groups were: Chow sedentary: CS; Chow exercise: CEX; HFD sedentary: HS; HFD NMN: HNMN; HFD exercise: HEX (12/group).

After 4 days of training from the age of 11 weeks, CEX and HEX mice underwent treadmill running 6 days (45 min/day) a week for 6 weeks using a Columbus Instruments Exer 3/6 Treadmill (0257-901M). Each session comprised a warm-up period of running at 3 m/min for 2 min then the speed was increased gradually to 15 m/min. In each session after 300 m, mice were rested by running slowly (6 m/min) for 3 min then returned to 15 m/min for another 20 min. Running of each mouse was confirmed visually and mice that stopped running by going on the stationary platform were gently pushed back onto the belt with a paper towel. Each exercise session was carried out an hour before the end of the light phase. All mice in the non-exercise groups experienced the treadmill with the belt turned off for 12 min, 5 days a week to control for environmental effects. NMN (Sigma N3501) was dissolved in PBS and injected i.p. daily 500 mg/kg body weight (Yoshino et al., 2011) for 17 days before sacrifice. Injection of NMN or vehicle (PBS) occurred daily at the end of the light phase. Exercised mice were rested a day before GTT and sacrifice.

### Glucose Tolerance Test

At 17 weeks of age animals were weighed and fasted for 5 h (7 am–12 pm). After the establishment of a baseline glucose level (Accu-ChekH glucose meter; Roche Diagnostics, Nutley, USA) mice were challenged with an i.p. glucose bolus (2 g/kg body weight). Blood glucose concentrations were measured at 15, 30, 60, 90, 120, and 180 min after glucose administration. NMN was injected 4 h before the glucose injection.

### Insulin

During the GTT 5 μl of blood was taken before, 15 min after and 30 min after glucose injection. Blood was pipetted with Drummond 5 μl Microcapillary tubes (Sigma–Aldrich) into an Ultra-Sensitive Mouse Insulin ELISA Kit plate (Crystal Chem Inc.) and the assay performed using the standard protocol.

### Sample Collection and Tissue Processing

At 18 weeks of age, after 5 h fasting, mice were deeply anesthetized (ketamine/xylazine 200/20 mg/kg, i.p.). NMN or vehicle was injected 4 h before anesthetic. The exercise group had their final session the day before cull. After measurement of naso-anal (N-A) length, a blood sample was collected by cardiac puncture. Mice were then sacrificed by decapitation. Brown adipose tissue (BAT), white adipose tissue (gonadal fat, retroperitoneal fat, inguinal fat), muscle (quadriceps, tibialis, soleus) were dissected and weighed, as well as organs (heart, liver). All tissue was snap frozen using liquid N_2_ and then stored at -80°C. Tissues were ground using a Tissue Pulverizer (Bessman) on dry ice and liquid N_2_.

### Triglyceride Assay

Ground liver tissue was homogenized in 1.5 ml of a chloroform–methanol (2:1) mixture using a Precellys 24 homogenizer (Bertin technologies, France) and transferred in a glass tube. Another 2.5 ml of chloroform-methanol mixture was added and samples were mixed for 20–22 h at room temperature on an electronic roller (BTR10-12V Ratek roller). After rolling, 2 mL of 0.6% NaCl was added; samples were vortexed and centrifuged (1,000 × *g*, 10 min, room temperature). The entire lower phase was transferred in to a new glass tube and evaporated under nitrogen gas in a heating block at 40°C for 40 min. The dried extract was dissolved in 150 μl of absolute ethanol. The triglyceride concentration was then measured using glycerol standard (Sigma, St. Louis, MO, USA). Liver triglyceride contents were determined using a colorimetric assay– TG reagent (Roche Diagnostics). Two hundred microliters of the reagent added with 10 μl of samples and standards in a 96 well plate. The plate was then incubated and gently shaken at 37°C for 6 min before reading with Bio-Rad iMark plate reader (Bio-Rad, Sydney, NSW, Australia).

### Protein Quantification

Proteins levels were quantified by using a Protein Assay Dye Reagent Concentrate (Bio-Rad). A prediluted Protein Assay standard Bovine Serum Albumin (BSA) set (Thermo Scientific) was used to make the standard curve.

### Citrate Synthase Assay

The assay was carried out according to the method described by Turner et al. (2007). Briefly, powdered tissue samples were homogenized 1:19 (wt/vol) in 50 mmol/l Tris-HCl, 1 mmol/l EDTA, and 0.1% Triton X-100, pH 7.2, using a polytron homogenizer (IKA T 10 Basic ultra-turrax, VWR instruments Pty Ltd) and were subjected to three freeze-thaw cycles with liquid N_2_. Citrate synthase, was determined at 30°C, using a Bio-Rad Imark microplate reader. Enzyme activities are presented as units per mg of protein, where units are defined as micromoles per minute.

### NAD^+^ Assay

Levels of NAD^+^ and its reduced form NADH were measured as previously described with modifications (Zhu and Rand, 2012). First, samples were homogenized in extraction buffer (10 mmol/l Tris/HCl, 0.5% Triton X-100, 10 mmol/l Nicotinamide, pH 7.4) and then centrifuged (12,000 × g for 5 min at 4°C), after which an aliquot of supernatant was taken for protein quantification. After phenol:chloroform: isoamyl alcohol (25:24:1) and chloroform extractions the supernatant was separated in two aliquots. One was used to measure total NAD. The other aliquot was acidified with HCl then neutralized with NaOH on ice to quantify NAD^+^. On a 96 well plate samples were mixed with alcohol dehydrogenase (ADH) in separate wells at room temperature. Total NADH and NAD^+^ were quantified using a Bio-Rad Imark microplate reader; data are presented as pmol of NAD^+^ or NADH per mg of protein.

### Western Blot

Protein was extracted as described previously Brandon et al. (2015). Approximately 30 mg of powdered tissue was homogenized in RIPA buffer (65 mmol/l Tris (pH 7.4), 150 mmol/l NaCl, 5 mmol/l EDTA, 1% Nonidet P-40, 0.5% sodium deoxycholate, 0.1% SDS, 10% glycerol, 1 μg/ml aprotinin, 1 μg/ml leupeptin, 10 mmol/l sodium fluoride, 1 mmol/l sodium vanadate, 1 mmol/l PMSF, and 50 mmol/l nicotinamide) using a Precellys 24 (Bertin Technologies). After lysis and homogenization the samples were incubated (4°C) for 2–3 h, then centrifuged at 12,000 × *g* to remove any insoluble particles and protein concentration was determined.

Clarified lysates were then diluted with 2X Laemmli buffer and heated to 65°C for 15 min. BLUeye Prestained Protein Ladder was used as molecular weight ladder. Equal amounts of protein (20 ug/well) were electrophoresed through a 4–15% precast gel (Criterion TGX, Bio-Rad) for 45 min at 150 V in running buffer (25 mmol/l Tris base, 192 mmol/l glycine, and 1% SDS, pH 8.3). Proteins were transferred via a semi dry transfer process with a Trans Blot Turbo System (Bio-Rad) onto PVDF membranes (Bio-Rad). Membranes were then blocked in 4% BSA in TBS-Tween for 1 h, then incubated overnight at 4°C with primary antibodies used at 1:1000 dilution; Mitoprofile total OXPHOS rodent antibody cocktail (MitoScience); PGC-1α (3G6) Rabbit mAb (Cell Signaling). The membrane was subjected to three 10 min washes with TBS-Tween, and incubation with appropriate secondary antibody (Cell Signaling) in 2% skim milk blocking solution in TBS-Tween at room temperature for 1 h, followed by three 10 min washes with TBS-Tween. For detecting bands, membranes were exposed to Clarity Western ECL Substrate (Bio-Rad) and visualized on a Bio-Rad ChemiDoc XRS. Membranes were stripped using Reblot Plus (10X) (Millipore) for 10 min at room temperature. Membranes were re-blocked and overnight at 4°C with GAPDH antibody (14C10) Rabbit mAb (Cell Signaling). The subsequent steps with this housekeeper blot were the same as described above for the OXPHOS cocktail and PGC-1α blots.

### Mitochondrial DNA Copy Number

Mitochondrial DNA copy number was measured by qPCR. DNA was extracted from muscle and liver. For this, 25–30 mg of tissue was subjected to lysis overnight (10 mmol/l Tris, 100 mmol/l NaCl, 10 mmol/l EDTA, 0.5% SDS pH8), containing proteinase K (1 μg/ul). The lysate was then mixed with phenol:chloroform and DNA extracted. Spectrophotometric quantification using Biospec-nano spectrophotometer (Shimadzu Biotech, Nakagyo-ku, Kyoto, Japan) determined DNA concentration and purity. A SYBR green Qpcr (SensiFAST SYBR, Bioline) was used to determine mitochondrial DNA copy number. Two primers were used, 36B4 (F = ACTGGTCTAGGACCCGAGAAG; R = TCAATGGTGCCTCTGGAGATT) for the nuclear genome (amplifies a region of the Rplp0 gene) and Cytb (F = CCCACCCCATATTAAACCCG; R = GAGGTATGAAGGAAAGGTATTAGGG) for the mitochondrial genome. Cytb and 36B4 levels were quantified with Roche LightCycler480 software, whereby standard curves were produced for each gene using templates generated by serial dilution of a sample made by combining an aliquot of DNA from each of the 60 samples. All sample PCRs were done in duplicate, normalization was done by dividing Cytb by 36B4.

### Quantitative RT-PCR

By using Tri-reagent (Sigma, St. Louis, MO, USA), RNA was extracted from 30 to 32 mg of tissue and stored at -80°C. RNA concentration and purity was determined (Biospec-nano spectrophotometer Shimadzu Biotech, Nakagyo-ku, Kyoto, Japan). One microgram of RNA was treated with DNase I Amplification Grade (Invitrogen; Cat# 18068015) and reverse transcribed to cDNA using an Omniscript Reverse Transcription kit (Qiagen, Valencia, CA, USA) following manufacturer’s instructions, and stored at -30°C. Expression of the following target genes: Sirt1 (F = TGTGAAGTTACTGCAGGAGTGTAAA; R = GCATAGATACCGTCTCTTGATCTGAA), Sirt3 (F = GGTTGAAGCTTATGGA, R = AGGTTTTGAGGCAGGGA), PGC-1a (F = TATGGAGTGACATAGAGTGTGCT, R = CCACTTCAATCCACCCAGAAAG), Cytb (Same primers as used for mtDNA copy number) was measured using the Roche LightCycler480. Standard curves were produced for each gene using templates generated by serial dilution of a combined cDNA sample from each of the 60 samples. All sample PCRs were done in duplicate, and all genes of interest were normalized by dividing by the geometric mean of two control genes Gapdh (F = AGGTCGGTGTGAACGGATTTG, R = TGTAGACCATGTAGTTGAGGT) and Ywhaz (F = GAAAATGAAGGGTGACTACTAC, R = CTGATTTCAAATGCTTCTTG) which had been determined with Normfinder software (MOMA) to be the most stable of 4 tested control genes (Hprt and Tbp being the other genes). No difference in expression of housekeeper genes was observed across treatment groups.

### Statistical Analysis

Results are expressed as mean ± SEM. All data were analyzed using one-way ANOVA, followed by *post hoc* LSD tests using SPSS. If data were not normally distributed they were log transformed to achieve normality before they were analyzed. Different superscripts represent significant differences between the designated groups (*Diet Effect; ^X^Exercise effect; ^∧^NMN effect). Significance levels are indicated; *p < 0.05, **p < 0.01, ***p < 0.001.

## Results

### High Fat Diet and Interventions Impacted Body Weight and Tissue Mass

**FIGURE 1:**
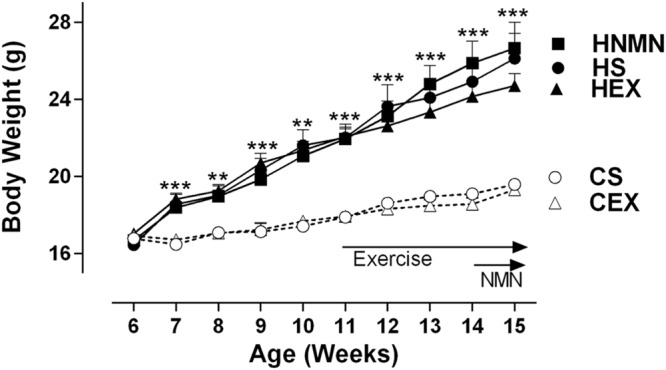
**Body weight of Chow sedentary (CS), Chow exercise (CEX), HFD sedentary (HS), HFD NMN (HNMN), and HFD exercise (HEX) mice over the experiment.** Data are shown as mean ± SEM (*n* = 11–12/group). Data were analyzed by one way ANOVA followed by LSD *post hoc* test. **P < 0.01, ***P < 0.001 significant difference HS compared to CS.

**FIGURE 2:**
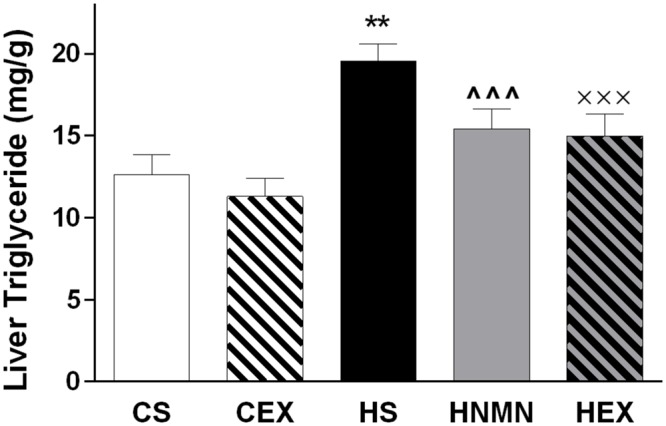
**Liver triglyceride (mg/g of tissue) of CS, CEX, HS, HNMN, and HEX mice.** Data are shown as mean ± SEM (*n* = 9–11/group). Data were analyzed by one way ANOVA followed by LSD *post hoc* test. **P < 0.01 significant difference HS compared to CS. ^∧∧∧^*P* < 0.001 significant difference HNMN compared to HS. ^XXX^*P* < 0.001 significant difference HEX compared to HS.

At 5 weeks of age, before separating the mice onto different diets (Chow or HFD), average body weight was 16.63 ± 0.12 g. A week later the mice selected for chow and HFD fed groups weighed 16.83 ± 0.81, and 16.71 ± 0.89 g, respectively. After 6 weeks of the diet, and before the exercise intervention started HFD fed animals were 23% heavier than chow-fed animals (21.94 ± 1.98 g vs. 17.90 ± 0.76 g). Increased weight gain by HFD fed animals continued until the end of the experiment (**Figure 1**; **Table 1**). We observed a slight (non-significant) reduction of the body weights of the exercised and NMN-treated mice (**Table 1**). There were no differences in tissue weights between the CS and CEX groups, either as net values or after correction for body weight. However, dissected fat pads, muscles and liver were significantly higher in HFD sedentary animals compared to those consuming chow. In those consuming HFD, both NMN and exercise interventions reduced net liver mass (**Table 1**) but this difference did not remain after correction for body weight. In the HEX group, exercise reduced net fat pad and liver mass compared to HS, and this was maintained for gonadal and inguinal pads when standardized by body weight. (**Table 1**). Quantitation of liver triglyceride revealed that the HFD increased liver triglyceride content by approximately 50%, and both exercise and NMN treatment significantly reduced liver triglyceride in the HFD-fed groups to around 20% above the level seen in mice consuming control diet (CS; **Figure 2**). This demonstrates that both interventions partially reversed the HFD-induced increases in liver triglyceride content. Exercise had no significant impact in chow fed mice (**Figure 2**).

**Table 1:** Dissected tissue weight and % body weight of Chow sedentary (CS), Chow exercise (CEX), HFD sedentary (HS), HFD NMN (HNMN), and HFD exercise (HEX) mice.

		CS	CEX	HS	HNMN	HEX
Final body weight (g)		19.5 ± 0.1	18.9 ± 0.3	28.2 ± 1.4***	25.9 ± 1.3	25.6 ± 0.8^xxx^
Liver (mg)		726.7 ± 39.0	753.3 ± 44.5	855.4 ± 35.8*	717.7 ± 26.3^∧∧^	755.7 ± 25.03^x^
Muscle (mg)	Quad	274.4 ± 5.3	277.5 ± 6.7	310.3 ± 6.3***	317.8 ± 9.2	328.6 ± 7.6
	AT	81.25 ± 4.09	80.25 ± 2.96	81.25 ± 3.34	89.583 ± 2.21	90.08 ± 3.89
	Soleus	13.92 ± 0.36	13.42 ± 0.40	16.33 ± 0.53**	15.50 ± 0.71	17.17 ± 0.55
WAT (mg)	Gonadal	150.5 ± 8.2	167.8 ± 11.2	1166.5 ± 172.4***	909.8 ± 157.5	754.3 ± 70.9^xx^
	Inguinal	156.5 ± 6.4	165.3 ± 12.1	728.25 ± 91.6***	580.4 ± 86.6	530.2 ± 44.5^x^
	RP	30.50 ± 2.06	32.83 ± 2.86	268.00 ± 49.04***	202.92 ± 32.33	186.33 ± 19.84^x^
Liver % BW		3.73 ± 0.20	3.98 ± 0.22	3.07 ± 0.12**	2.82 ± 0.14	2.98 ± 0.14
Muscle % BW	Quad	1.41 ± 0.03	1.48 ± 0.04	1.12 ± 0.07***	1.25 ± 0.05	1.29 ± 0.04
	AT	0.42 ± 0.02	0.43 ± 0.02	0.29 ± 0.02***	0.36 ± 0.02	0.35 ± 0.01^x^
	Soleus	0.07 ± 0.001	0.07 ± 0.002	0.06 ± 0.003**	0.06 ± 0.002	0.07 ± 0.002
WAT % BW	Gonadal	0.77 ± 0.04	0.89 ± 0.05	3.92 ± 0.47***	3.27 ± 0.43	2.92 ± 0.19^xxx^
	Inguinal	0.80 ± 0.03	0.87 ± 0.06	2.49 ± 0.23***	2.12 ± 0.23	2.04 ± 0.12^xxx^
	RP	0.16 ± 0.01	0.17 ± 0.01	0.89 ± 0.13***	0.74 ± 0.08	0.71 ± 0.06^xxx^

Data are shown as mean ± SEM (*n* = 9–11/group). Data were analyzed by one way ANOVA followed by LSD *post hoc* test. *P < 0.05, **P < 0.01, ***P < 0.001 significant difference HS compared to CS. ^∧∧^*P* < 0.01 significant difference HNMN compared to HS. ^X^*P* < 0.05, ^XX^*P* < 0.01, ^XXX^*P* < 0.001 significant difference HEX compared to HS.

### NMN Supplementation and Exercise Improved Glucose Tolerance in HFD Fed Mice

**FIGURE 3:**
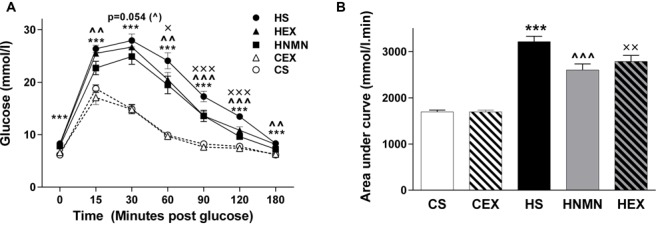
**Glucose tolerance test **(A**, mmol/l) in CS (open circle) CEX (open triangle), HS (closed circle), HEX (closed triangle) and HNMN (closed square) mice, and **(B)** Area under the curve (mmol/l min).** Data are shown as mean ± SEM (*n* = 11–12/group). Data were analyzed by one way ANOVA followed by LSD *post hoc* test. ***P < 0.001 significant difference HS compared to CS. ^∧∧^*P* < 0.01, ^∧∧∧^*P* < 0.001 significant difference HNMN compared to HS. ^X^*P* < 0.05, ^XX^*P* < 0.01, ^XXX^*P* < 0.001 significant difference HEX compared to HS.

Glucose tolerance test results shown in **Figure 3** demonstrate that both interventions improved HFD-induced glucose intolerance. The basal glucose concentration and time taken to clear injected glucose was significantly higher in HFD sedentary animals compared to chow fed animals (**Figure 3A**). The HEX and HNMN groups had reductions in plasma glucose concentration compared to HS from 15 min after glucose injection until 180 min. However, there was no difference in the GTT between the CS and CEX groups. The beneficial effects of NMN and exercise in increasing the rate of glucose clearance from the blood of obese mice were also significant when the area under the curve was assessed (**Figure 3B**, both *P* < 0.01). To test insulin concentrations under fasting conditions or after a glucose bolus we measured plasma insulin during the GTT. High fat diet fed animals showed significantly higher plasma insulin concentrations than chow fed animals; neither exercise nor the NMN intervention had any impact on insulin concentrations during the GTT (**Supplementary Figure S1**). This may suggest that the insulin peak occurred prior to the 15 min time-point.

### NAD^+^ and NADH Levels Are Altered by HFD but Effects of Interventions Are Tissue Specific

**FIGURE 4:**
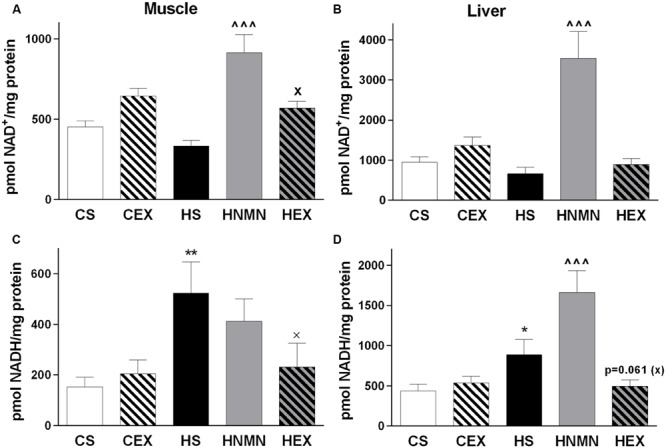
**NAD^+^**(A)** and NADH **(B)** content (pmol/mg protein) of quadriceps muscle and liver **(C,D)** of CS, CEX, HS, HNMN, and HEX mice.** Data are shown as mean ± SEM (*n* = 6–8/group). Data were analyzed by one way ANOVA followed by LSD *post hoc* test. *P < 0.05, **P < 0.01 significant difference HS compared to CS. ^∧∧∧^*P* < 0.001 significant difference HNMN compared to HS. ^X^*P* < 0.05 significant difference HEX compared to HS.

NAD^+^ levels were measured in both muscle and liver. Previously it was shown that 7 days of NMN injection increased NAD^+^ levels in livers of diabetic mice (Yoshino et al., 2011), and exercise increased NAD^+^ in rodent skeletal muscle (Cantó et al., 2010; Koltai et al., 2010). In our experiment there was a trend for a reduction in NAD^+^ by HFD but a significant increase by NMN injection in both tissues, with the greatest increase in liver (**Figures 4A,B**). Exercise also increased NAD^+^ levels in muscle but not in liver compared to the HFD sedentary group. In muscle and liver, NADH was significantly increased by HFD in both tissues, and this was ameliorated by exercise in both tissues (significantly in muscle; **Figures 4C,D**). In liver the HNMN group had a further significant increase of NADH (**Figure 4D**), possibly due to the extremely high levels of reduced and oxidized NAD in the tissue.

### Citrate Synthase Activity Was Reduced by HFD but Increased by Interventions in a Tissue-Specific Manner

**FIGURE 5:**
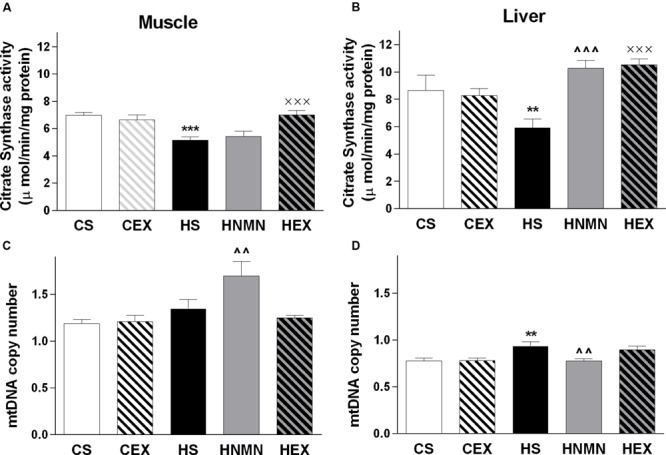
**Citrate synthase (μ mol/mg protein) and mtDNA copy (arbitrary number) in quadriceps muscle **(A,C)** and liver **(B,D)** of CS, CEX, HS, HNMN, and HEX mice.** Data are shown as mean ± SEM (*n* = 9–11/group). Data were analyzed by one way ANOVA followed by LSD *post hoc* test. **P < 0.01, ***P < 0.001 significant difference HS compared to CS. ^∧∧^*P* < 0.01, ^∧∧∧^*P* < 0.001 significant difference HNMN compared to HS. ^XXX^*P* < 0.001 significant difference HEX compared to HS.

We measured citrate synthase activity in both muscle and liver (**Figures 5A,B**). In muscle, HFD consumption led to a reduction in citrate synthase activity, which was ameliorated by exercise but not by NMN. In liver, there was also a trend for reduced citrate synthase activity due to HFD associated obesity (*P* = 0.086) but both exercise and NMN led to significant increases relative to the HFD vehicle group (**Figure 5B**, both *P* < 0.001).

### Tissue-Specific Effects of NMN Supplementation on Mitochondrial DNA Copy Number

To investigate mitochondrial biogenesis, we measured mitochondrial DNA (mtDNA) copy number in muscle and liver (**Figures 5C,D**). Consumption of HFD significantly increased copy number in liver, and there was a similar trend in muscle (*P* = 0.087). There were contrasting effects of NMN treatment in muscle and liver of the HFD mice as mtDNA copy number was increased in the former and decreased in the latter.

### OXPHOS Proteins and mRNA Levels of Mitochondria Associated Genes

Five different complexes are involved in oxidative phosphorylation in the mitochondria (Huss and Kelly, 2005). We measured the total levels of a representative protein from complexes I, II, III, and V. In muscle we found no changes in proteins from complexes I, II, and V due to HFD or the two interventions. However, complex I and II proteins were increased in CEX compared to CS mice (**Supplementary Figure S2**). In liver, there were no diet-induced changes in complexes I, II, III, and V. However, exercise in obese mice decreased the levels of complexes II and III proteins (**Supplementary Figure S2**). PGC-1α is a master regulator of mitochondrial function and biogenesis. However, we did not detect any differences in total level of the protein between groups (**Supplementary Figure S2**).

We also assayed mRNA levels of genes that are responsible for mitochondrial biogenesis, mitochondrial content and function. No HFD or exercise or NMN intervention-induced transcriptional changes were seen in Sirt1, Sirt3, PGC-1α or Cytb (**Supplementary Figure S3**).

## Discussion

There is much current interest in evaluating the utility of drugs that increase NAD^+^ levels. These drugs include resveratrol (Baur et al., 2006; Lagouge et al., 2006; Timmers et al., 2011) NMN (Yoshino et al., 2011; Canto and Auwerx, 2012) and nicotinamide riboside (NR; Canto and Auwerx, 2012; Gariani et al., 2016). Here we compared the effects of NMN and exercise in the context of dietary obesity. Our study comprised a relatively short i.p. administration of NMN (17 days), once obesity was established in the mouse, and like the exercise intervention we designed, it did not have a significant effect on body weight. Yoshino et al. (2011), also reported no body weight changes in their 7–10 days NMN treatment of HFD-fed mice (Yoshino et al., 2011). We predict that longer term NMN supplementation would reduce body weight in mice as this was observed with longer term (beyond 7 weeks) food supplementation of NR (Canto and Auwerx, 2012; Gariani et al., 2016) or resveratrol (Baur et al., 2006; Lagouge et al., 2006). The exercise intervention chosen for our study was a relatively mild regime. However, both NMN and exercise induced considerable physiological and metabolic changes that are consistent with amelioration of obesity-related phenotypes. Furthermore the outcomes give insight into the primary effects of NMN supplementation as opposed to secondary benefits such as those caused by weight loss. Finally, our study is the first in the area of NAD^+^ therapy to perform a head-to-head comparison of an NAD^+^-increasing drug and exercise. Interesting differences emerged between these two interventions when we examined the underlying markers of mitochondrial biology in muscle and liver.

Many studies have described changes in mitochondrial parameters due to HFD-induced obesity (Koves et al., 2008; Begriche et al., 2013). However, the extent and even direction (increases or decreases) of those changes vary. This variation is due to a multitude of factors such as differences in the organism, tissue type, formulation of diet, length of diet, level of tissue adiposity/inflammation, and intensity of exercise. In general, muscle responds to a HFD by increasing mitochondrial content, and thereby its capacity to catabolise fats. However, the mitochondria can have compromised function due to overload which is thought to be induced by excessive beta-oxidation (Koves et al., 2008). In severe fatty liver disease liver mitochondrial function is reduced through a variety of mechanisms reviewed in Begriche et al. (2013). However, in fatty livers without cirrhosis mitochondrial biogenesis and function may be increased or decreased. Variation in the factors listed above is thought to be responsible for this inconsistency (Begriche et al., 2013).

Exercise increases the rates of lipolysis and fat oxidation (Turner et al., 2014) and accordingly exercise training in obese rodents has been shown to increase lipid metabolism and mitochondrial biogenesis in muscle (Suga et al., 2013; Kwon et al., 2014). Fewer studies have examined liver mitochondria in exercise trained rodents. Exercise training in non-obese rodents has been shown to increase mitochondrial respiration (Sun et al., 2010; Fletcher et al., 2014) but both increases (Sun et al., 2010) and decreases (Lu et al., 2013) in mitochondrial biogenesis have been reported.

As expected, obesity impacted the levels of NAD^+^ and NADH in the muscle and liver. The increase in NADH in both of these tissues is indicative of a diminished ability of the mitochondria to generate ATP. NMN supplementation increased NAD^+^ levels to a greater degree in liver than muscle, suggesting a greater impact of NMN supplementation in the liver.

In muscle from obese mice, exercise increased NAD^+^, and decreased its reduced form, NADH, suggesting an improvement of cellular oxidative capacity. However, in NMN-supplemented mice the concomitant high levels of NAD^+^ and NADH may be an indicator of high levels of reduction of NAD^+^ to NADH. The significant decrease in liver mass and triglyceride content, and increased CS activity of HFD-fed mice following NMN-supplementation suggest another aspect of the NMN intervention is likely to be increased catabolism of fats.

Despite the large impact on markers of mitochondrial function and fat deposition in the livers of NMN-supplemented obese mice, other mitochondrial markers were less affected. Compared to the HS group mtDNA copy number was slightly decreased in HNMN livers and no changes were detected in the abundance of proteins involved in oxidative phosphorylation. Canto and Auwerx (2012) and Gariani et al. (2016) observed that long-term NR-supplementation also decreased liver weight, liver fat content, increased NAD^+^ levels and CS activity. However, unlike our study they also reported an increase in mtDNA and OXPHOS complex V (ATP Synthase). This suggests that a major clinical feature of NAD^+^-increasing drugs, namely reduction of liver fat, does not require an increase in mtDNA and OXPHOS components. If this is the case, then the NAD^+^-stimulated fat metabolism pathways (Han et al., 2010; Ponugoti et al., 2010) may be more clinically important than NAD^+^-stimulated mitochondrial biogenesis (Wu et al., 1999; Nemoto et al., 2004) pathways in liver. However, we cannot rule out the possibility that species differences, the type of NAD^+^ precursor, or mode of delivery may also account for this discrepancy.

In contrast to liver, muscle NMN supplementation was associated with an increase in mtDNA, though no changes were seen in CS activity or OXPHOS protein levels. Canto et al., (Canto and Auwerx, 2012) also reported increased mtDNA in quadriceps with long-term NR supplementation (incidentally also with no changes to Sirt1 or PGC-1α transcript levels), however, they also observed increases in OXPHOS complex V. These results suggest that NAD^+^-increasing drugs have different effects on the muscle from the liver. More specifically, that mitochondrial DNA content is more stimulated in the muscle than the liver. The absence of an OXPHOS complex V increase in our model may be due to the short-term of intervention or again due to differences in drug-type or delivery across studies. Other markers of mitochondrial function such as substrate utilization would have been useful to investigate this further.

The exercise intervention had a greater impact on the HFD fed mice than the control diet group, the CEX mice were similar to the CS group across many parameters. For example there were no differences between CS and CEX in body or organ weights, GTT, NAD^+^, or NADH levels, CS activity or mtDNA. The only differences were an increase in OXPHOS complex I and II proteins in the muscle of the CEX group. This small difference is likely due to the exercise intervention being relatively mild, and both groups being young healthy mice. The intervention did however, have a considerable impact in the HFD group (HEX) compared to the HFD sedentary group (HS). Although there was no significant difference in body weight, white adipose tissue weights were reduced. Other measurements such as GTT, liver weights and liver triglyceride levels were improved in HEX to very similar levels as in the HNMN intervention group. This similarity increases the value of the comparison between the two interventions as, for example, we can contrast the mitochondrial parameters in the livers of the two groups that ultimately resulted in similar reduction in triglyceride levels compared to the HS group (discussed below).

In quadriceps muscle the HEX groups showed effects that were consistent with long-term exercise (Brandauer et al., 2013; Yuan et al., 2014). More NAD^+^ was produced, presumably through increased catabolism of fat and carbohydrates resulting in oxidation of NADH. This is supported by the increase in CS activity in HEX vs. HS muscle. The NAD^+^ and NADH changes in the HEX and HNMN groups were similar (though NADH reduction in HNMN was not significant) which suggests that in that regard NMN-supplementation does mimic exercise. However, CS activity was increased by exercise but not NMN which points to some underlying differences in mitochondrial function. Mitochondrial biogenesis may also differ in muscle between the two interventions as exercise did not increase mtDNA, while NMN did.

The exercise effects in liver in the HEX compared to the HS group were consistent with those seen with long-term exercise (Goncalves et al., 2013). The largest difference between the two interventions in the liver was in the level of NAD^+^ and NADH, as NMN-supplementation increased them considerably, while exercise did not. This is in stark contrast to the similarities in triglyceride content, CS activity and mtDNA copy number. Investigation of this difference is likely to be central to future work that aims to explain how liver mitochondrial biology is differentially regulated by exercise and NAD^+^-increasing drugs. For example NMN-supplementation may stimulate mitochondrial function though saturating the cell with an oxidizing agent (NAD^+^), or through stimulating sirtuin mediated pathways (Kim ref), while the effects of exercise on liver mitochondria may be more regulated by signalzing from muscles or the brain (Lima et al., 2013).

## Conclusion

In summary, in our study both exercise and NMN were shown to partly ameliorate the pathophysiology of HFD-induced obesity in female mice. However, the two interventions have tissue-specific differences in their alteration of mitochondrial biogenesis and function. These differences have implications for the potential use of NMN for the treatment of obesity or fatty liver. Given that NMN treatment resulted in greater changes in markers of mitochondrial function in liver than muscle, NAD precursor-based therapies may be more effective for treatment of obesity-associated liver diseases such as non-alcoholic fatty liver disease.

## Author Contributions

Conceived and designed the experiments: MM, NY, and DS. Performed the experiments: GU and NY. analyzed the data: GU, NY, and MM. Wrote the paper: GU, NY, DS, and MM. All authors reviewed and approved the final manuscript.

## Conflict of Interest Statement

DS is a consultant to and inventor on patents licensed to Ovascience, Metrobiotech, and GlaxoSmithKline. All the other authors declare that the research was conducted in the absence of any commercial or financial relationships that could be construed as a potential conflict of interest.

## Supplementary Material

FIGURE S1 **Insulin concentrations during GTT of Chow sedentary (CS), Chow exercise (CEX), HFD sedentary (HS), HFD NMN (HNMN), and HFD exercise (HEX) mice, expressed relative to CS concentration at time 0**. Data are shown as mean ± SEM (*n* = 11–12/group) and are expressed relative to CS at baseline. Data were analysed by one way ANOVA followed by LSD *post hoc* test. ***P* < 0.01, ****P* < 0.001 significant difference HS compared to CS.

FIGURE S2 **Mitochondrial complexes (I, II, V) in muscle **(A–C)**, PGC-1a protein levels in muscle **(D)** and Mitochondrial complexes (I, II, III, V) in liver **(E–H)** of CS, CEX, HS, HNMN, and HEX mice**. Data are shown as mean ± SEM (*n* = 7–8/group). Data were analyzed by one way ANOVA followed by LSD *post hoc* test. **P* < 0.05, ***P* < 0.01. Significant difference HS compared to CS; ^X^*P* < 0.05 significant difference HEX compared to HS.

FIGURE S3 **mRNA expression of targeted genes in muscle Sirt1 **(A)**, Sirt3 **(B)**, PGC-1a **(C)**, and CYTB **(D)** of CS, CEX, HS, HNMN, and HEX mice**. Data are shown as mean ± SEM (*n* = 8–11/group). Data were analyzed by one way ANOVA followed by LSD *post hoc* test.
