# Supplementary figures and images for: Head to Head Comparison of Short-Term Treatment with the NAD+ Precursor Nicotinamide Mononucleotide (NMN) and 6 Weeks of Exercise in Obese Female Mice
Source: Front Pharmacol. 2016 Aug 19;7:258. doi: 10.3389/fphar.2016.00258 (PMC4990541; doi:10.3389/fphar.2016.00258)

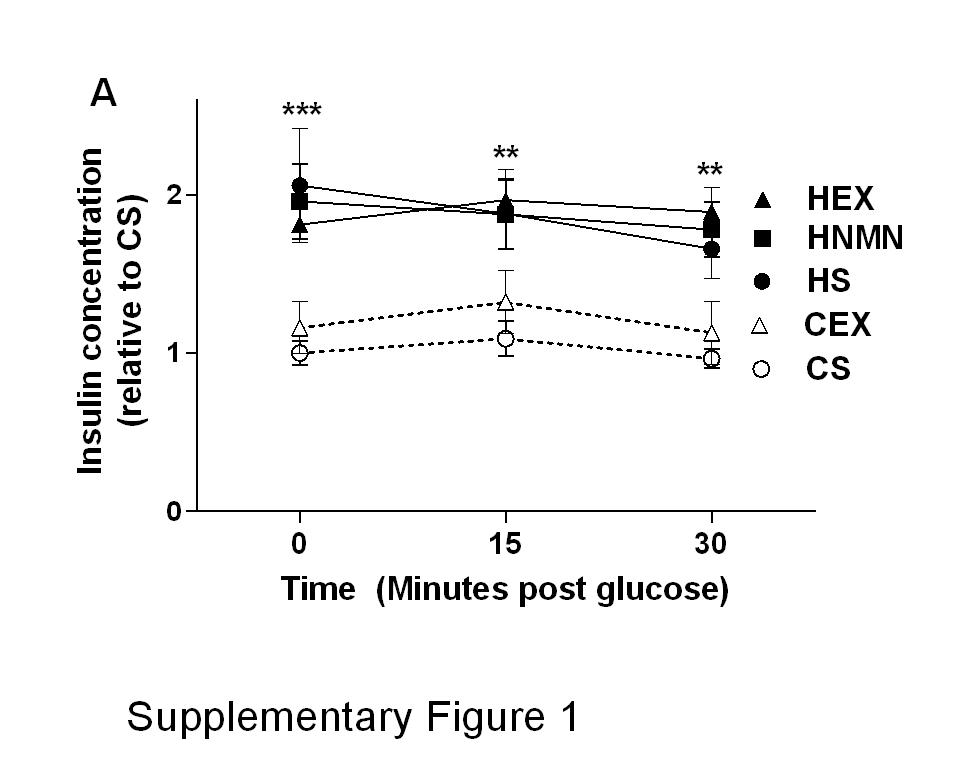

Supplement: FIGURE S1 — Insulin concentrations during GTT of Chow sedentary (CS), Chow exercise (CEX), HFD sedentary (HS), HFD NMN (HNMN), and HFD exercise (HEX) mice, expressed relative to CS concentration at time 0. Data are shown as mean ± SEM (n = 11–12/group) and are expressed relative to CS at baseline. Data were analysed by one way ANOVA followed by LSD post hoc test. **P < 0.01, ***P < 0.001 significant difference HS compared to CS. [file Image_1.TIF]

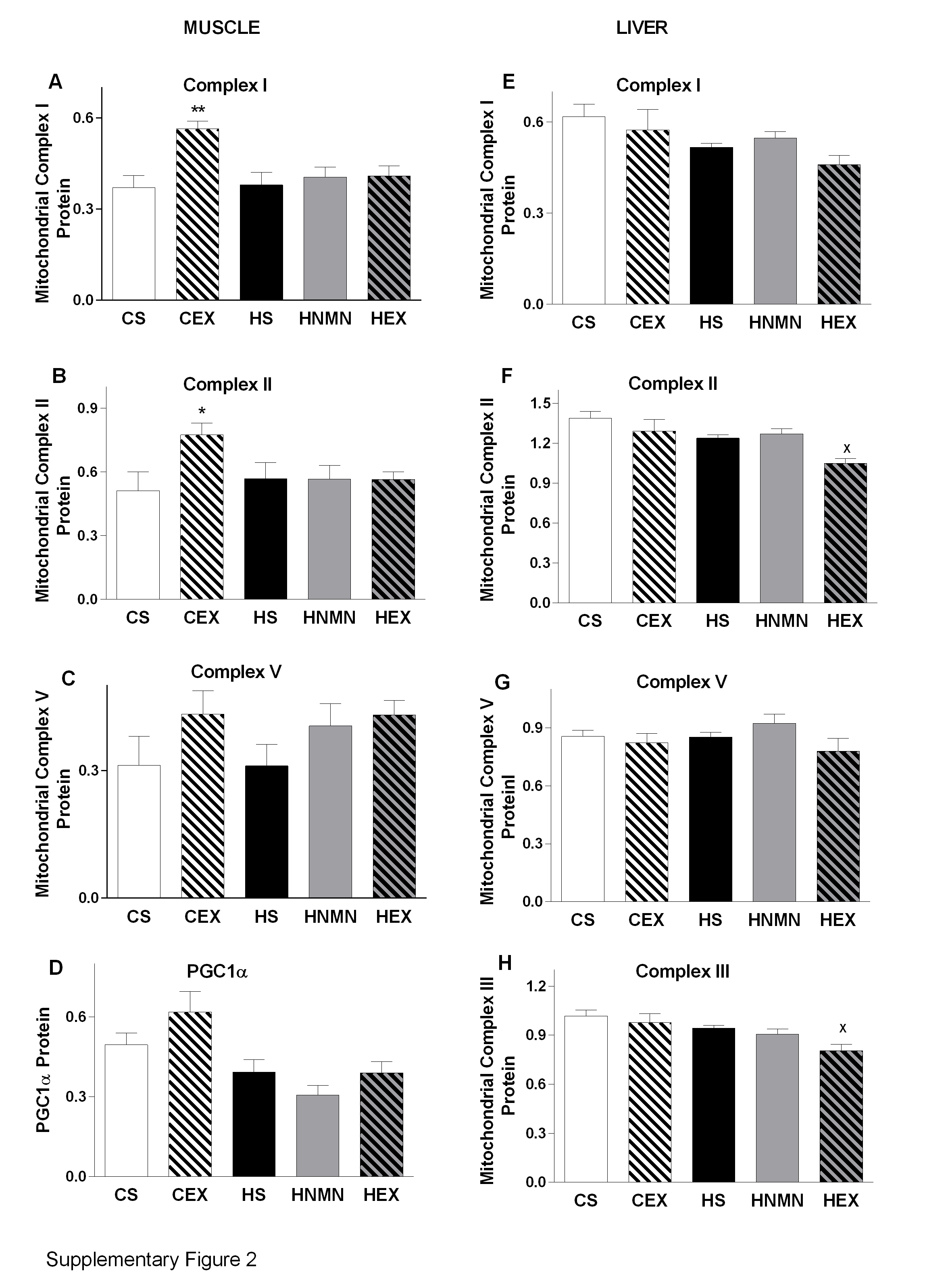

Supplement: FIGURE S2 — Mitochondrial complexes (I, II, V) in muscle (A–C), PGC-1a protein levels in muscle (D) and Mitochondrial complexes (I, II, III, V) in liver (E–H) of CS, CEX, HS, HNMN, and HEX mice. Data are shown as mean ± SEM (n = 7–8/group). Data were analyzed by one way ANOVA followed by LSD post hoc test. *P < 0.05, **P < 0.01. Significant difference HS compared to CS; XP < 0.05 significant difference HEX compared to HS. [file Image_2.TIF]

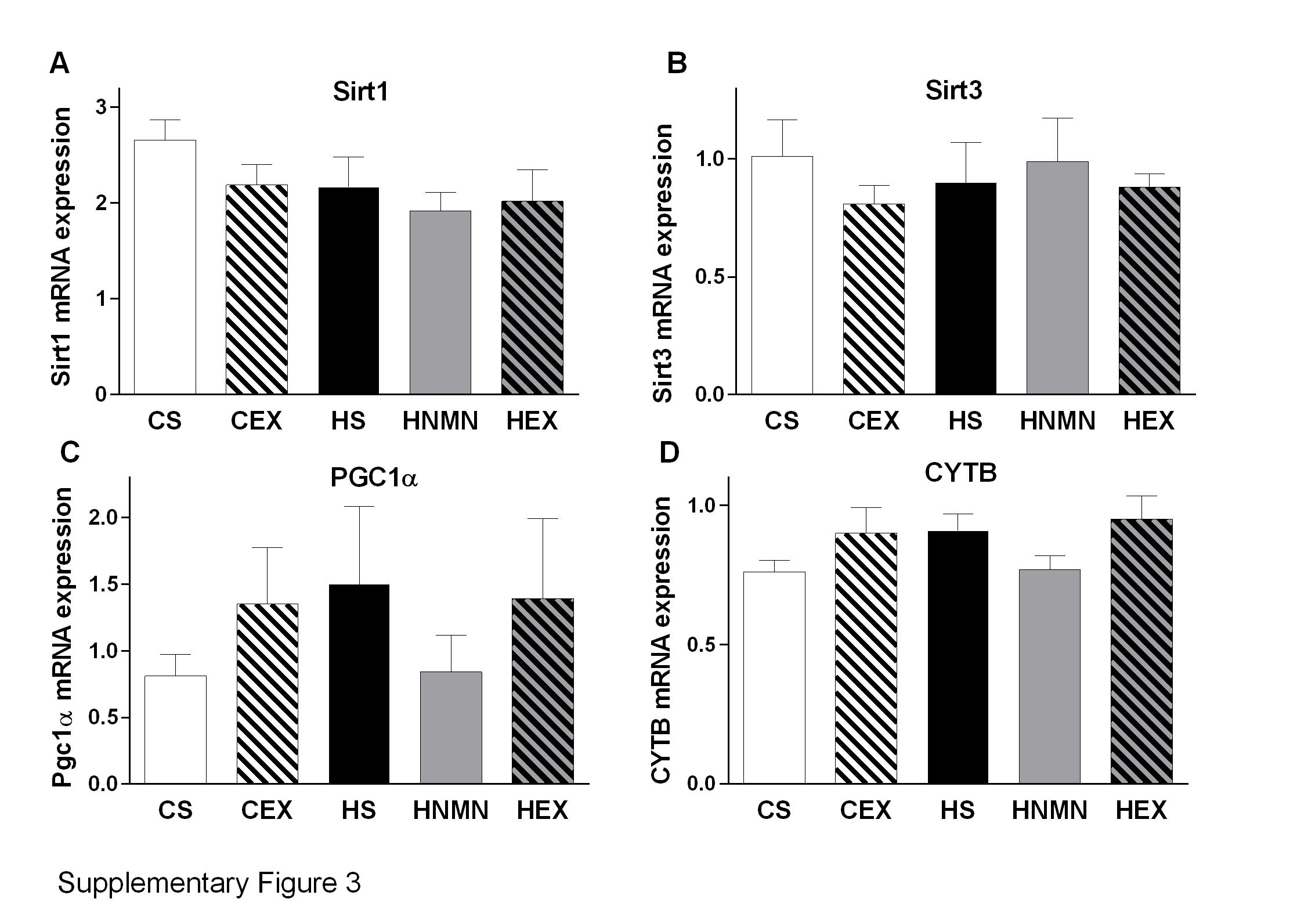

Supplement: FIGURE S3 — mRNA expression of targeted genes in muscle Sirt1 (A), Sirt3 (B), PGC-1a (C), and CYTB (D) of CS, CEX, HS, HNMN, and HEX mice. Data are shown as mean ± SEM (n = 8–11/group). Data were analyzed by one way ANOVA followed by LSD post hoc test. [file Image_3.TIF]
